# Barriers with Valve Mechanisms Are Predicted to Protect Crops from Slug Carriers of Rat Lungworm Disease

**DOI:** 10.3390/pathogens12060847

**Published:** 2023-06-19

**Authors:** Genevieve C. Pang, Amy T. Hou, Ryan Tamashiro, Kristin M. Mills, Lorrin W. Pang

**Affiliations:** Hawaii State Department of Health, Wailuku, HI 96793, USA; amy.hou.nsw@doh.hawaii.gov (A.T.H.); lorrin.pang@doh.hawaii.gov (L.W.P.)

**Keywords:** Angiostrongyliasis, *Angiostrongylus cantonensis*, *Parmarion martensi*, rat lungworm disease, valve effect, barrier, deterministic model, crops

## Abstract

Angiostrongyliasis (Rat Lungworm disease) is an emerging parasitic disease caused by the ingestion of gastropods infected with the neurotropic nematode *Angiostrongylus cantonensis*. The reduction of crop infestation with infected slug carriers may vary widely by protection method. We explored the application of barriers with valve mechanisms, whereby selective directional forces caused a greater number of slugs to exit than enter the protected plot, leading to decreased slug population densities at a steady state. Using field data, we constructed predictive models to estimate slug population densities at a steady state in protected plots with (1) no valve effect, (2) a valve effect, (3) no valve effect with a single breach of the barrier, (4) a valve effect with a single breach of the barrier, (5) a valve effect with a constant breach of the barrier, and (6) a repelling effect. For all scenarios, plots protected using a barrier with a valve effect had consistently lower slug densities at a steady state. Our findings support the use of barriers with valve mechanisms under different conditions, and potentially in combination with other interventions to reduce the contamination of crops by slug carriers of *A. cantonensis*. Improving barriers extends beyond disease mitigation to economic and cultural impacts on the local farmer and consumer communities.

## 1. Introduction

Angiostrongyliasis (rat lungworm disease) is an emerging parasitic disease caused by the neurotropic nematode *Angiostrongylus cantonensis*, which uses gastropods (i.e., snails and slugs) as intermediate hosts and rats as definitive hosts to complete its life cycle. This disease was discovered in southern China in the 1930s and has since spread widely throughout Southeast Asia, Japan, Australia, South America, Southeastern United States, and several island chains, including the Caribbean and Hawaii. The wide distribution of this disease can be attributed, in part, to the proximity of carrier snails and slugs to human habitations and farms, in addition to the rapid and ubiquitous dispersal of rat hosts.

Humans can become accidental hosts when they ingest produce containing uncooked or partially cooked slugs that are infected with juvenile stages of *A. cantonensis*. In humans, larvae die upon reaching the central nervous system, causing a significant inflammatory response that can result in neurologic symptoms and eosinophilic meningitis [1]. Treatment options for this disease are limited, with some evidence for the effectiveness of anthelminthics and corticosteroids [2,3,4].

Over the past two decades, Hawaii state has experienced an increased incidence of rat lungworm disease. This pathogen has been detected on five of the six most inhabited islands (Oahu, Maui, Hawaii, Kauai, and Lanai), with infection prevalence of gastropods estimated for Kauai, Hawaii, Maui Nui (including Maui, Lanai, and Molokai), and Oahu at 34%, 33%, 18%, and 10%, respectively [5]. This epidemic has been spurred by the documented invasion in 2004 of *Parmarion martensi* [6], a semi-slug that inhabits peridomicile settings, which has since become the primary gastropod carrier of *A. cantonensis* in Hawaii state [7,8]. Although *P. martensi* had been established as the primary carrier species of *A. cantonensis* in Hawaii, a 2014 survey identified a total of 16 carrier gastropod species, some with an infection prevalence approaching 30% [8]. Furthermore, among-island differences in host and pathogen subcommunities likely contribute to observed differences in the carrier species at local scales. For example, in Maui, a recent survey for samples collected from 2016 to 2017 brought the new total of carrier species in Hawaii state to 21, with a higher infection prevalence estimated for *Deroceras reticulatum* (50%) than *P. martensi* (31%) [9]. Therefore, various slug species may contribute to the spread of this disease within Hawaii state, requiring monitoring and control measures of all potential carrier species for a given island. Mitigation strategies include public education efforts on best practices for preparing produce [10], active monitoring of sentinel species (e.g., hind-leg paralysis in juvenile dogs), and the application of barriers or poisoned bait to reduce the number of snails and slugs infesting crops.

Historically, barriers with valve effects have been used to both amplify and reduce the population densities of organisms. A valve influences the direction of animal movement and can increase or decrease the density of animals on either side of the barrier. This directionality is essential to maintain spatial differences in density at a steady state [11]. Barriers to slugs may apply this valve design, whereby slugs can more easily leave than enter a protected area (Table 1). The time to reach a steady state may be affected directly by the size and shape of the internal area and rate of animal movements, and indirectly by population dynamics (e.g., births and deaths) and seasonality (e.g., high or low season) of the external population [12].

An apparatus with a novel valve design was previously created and tested in a laboratory and field study to combat rat lungworm disease slug carriers [13]. In a laboratory setting, *P. martensi* was observed to readily climb vertical surfaces of a multitude of barrier materials. However, the addition of electrified wires placed on the outside of the vertical fence surrounding a crop created a valve effect; entering slugs were shocked, and either retreated or fell back outside, and slugs inside the protected area that exited over the top of the fence were shocked and fell across the barrier to the outside [13]. This combination of electricity and gravity created a selective directional force such that the overall number of slugs exiting the protected plot was greater than those entering. In the field study, after approximately three weeks, the internal population density of the dominant local species, *D. reticulatum*, was reduced by 90% in the protected plot at a steady state [13]. During the field study, researchers noted an unexpected breach of the barrier, whereby vegetation cuttings served as a convenient bridge over the wall; the resulting spike in the internal slug density returned to a steady state in approximately one week [13]. This observation indicated that *P. martensi* dispersion occurred on a much shorter timescale than reproduction and death, suggesting that birth and death rates may be excluded from future predictive models of valve effects, following the assumptions of movement ecology [14,15].

To explore how barriers with valve effects may reduce slug population densities in protected areas, we constructed a predictive model based on previous invasion models that mechanistically described organismal movement patterns in response to barriers [16]. We used findings from a previous field experiment to estimate the effect of a barrier apparatus with a valve mechanism on slug population densities [13], as well as laboratory experiments from the literature for slug speeds from which to estimate the velocity used in these models [12] (see Appendix A). We investigated the valve mechanism behind previously observed differences between the densities of internal and external slug populations at steady state. We also used these models to address practical questions posed by farmers facing habitat-specific challenges causing regular or irreparable barrier breaches. This model can be used to explore different scenarios, as well as predict how new unforeseen environmental conditions factor into controlling slug populations and reducing human disease.

## 2. Materials and Methods

To explore the real-world applications of slug barriers with valve mechanisms, we compared the internal and external slug population densities at a steady state under different conditions. We included six possible scenarios for slug control and their effects on the resulting populations at a steady state: (1) no valve effect, (2) a valve effect, (3) no valve effect and single breach of the barrier, (4) a valve effect and single breach of the barrier, (5) a valve effect and various levels of a constant breach of the barrier, and (6) a repelling effect. We built a deterministic model for each scenario using parameter estimates from previous fields and laboratory findings [13] using R programming language [17].

These scenarios are presented in order of complexity, with comparisons made to previous scenarios. Scenarios 1 and 2 investigate the internal population density at a steady state of plots without and with an added valve effect favoring exit over entry, respectively. Scenarios 3 and 4 investigate the resulting internal population density after a temporary breach of barriers without and with an added valve effect. Scenario 5 investigates the effect of a barrier with a valve effect when there is a constant breach of the barrier, allowing slugs to travel between populations on either side of the barrier. Scenario 6 investigates a repelling effect (i.e., barrier materials that reduce slug crossing, such as a zone of salt, diesel oil, copper, or an electric barrier laid horizontally). All scenarios assume the direction of slugs to be random.

In scenarios 1,2,3, and 4, we used the following mathematical equation to determine *y*(*t*), the internal slug population as a function of time. We referred to *D* as the external population density, which was assumed to be constant to allow for relative comparisons of barrier effects with valve mechanisms [13]. The terms *P* and *Q* represented the proportion of slugs crossing the barrier (entering and exiting) once they reached it, respectively. This equation contained the constant *K*, which adjusted the baseline slug population in the protected plot. We defined *C* as the circumference of the barrier, *V* as the vector of slug speed and direction, *A* as the area of the protected plot, and *t* as time.

$$y\left(t\right)=D\left(\frac{P}{Q}\right)+Ke^{-\left(\frac{CVQ}{A}\right)t}(\mathrm{Scenarios}\;1,2,3,4,6)$$

**Figure 1:**
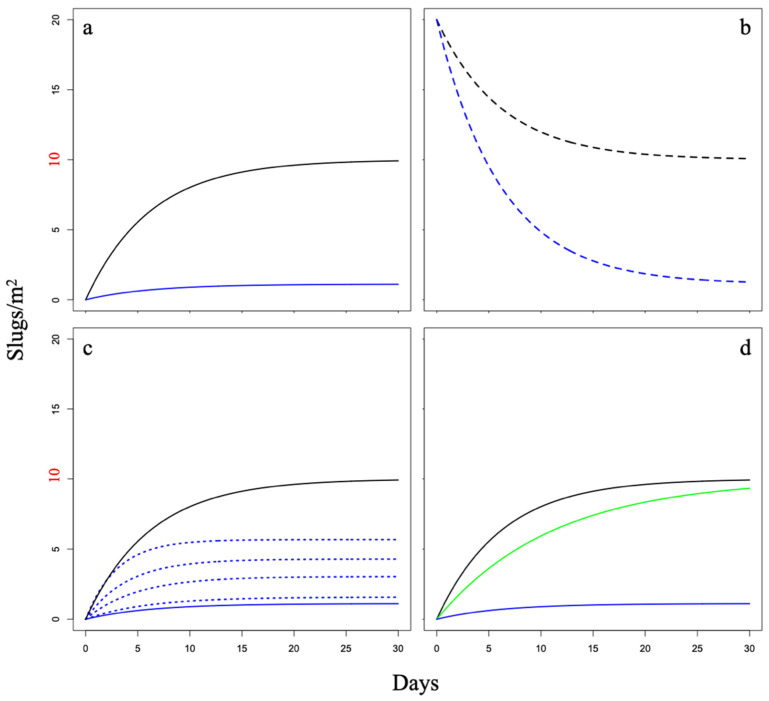
Internal population densities in protected plots in (**a**) scenarios 1 (without a valve effect) (black) and 2 (with a valve effect) (blue). Internal population densities in (**b**) scenarios 3 (without a valve effect) (black) and 4 (with a valve effect) (blue) after a single barrier breach, where the internal density was initially raised to 200% of external density. Internal population densities (**c**) without a valve effect (solid black), with a valve effect (solid blue), and in scenario 5 with four levels (5%, 25%, 50%, and 95% the size of the barrier circumference) of barrier breaches allow a constant flux of slugs between the internal and external populations (dotted). Internal population density (**d**) without a valve effect (black), with a valve effect (blue), and in scenario 6 with barrier materials creating a repelling effect (green) that slows the rate at which the internal population reaches its steady state. For all scenarios, the external population density was set to 10 slugs/m^2^ (red).

The *P/Q* term represented the ratio of the number of slugs entering to exiting the protected plot once they reached the barrier. In future models, this ratio may be modified to affect the strength and direction of the valve effect. In scenario 1 (no valve effect), *P*/*Q* = 1, as the ratio of slugs entering and exiting via the barrier was equal (Figure 1a). In scenario 2 (valve effect), *P* < *Q*, as a smaller ratio of slugs entered via the barrier, than exited (Figure 1a). We modified *K* to explore a single breach scenario without (scenario 3) and with (scenario 4) a valve effect. We explored a single breach at the start of the experiment that made the internal population density twice that of the external population immediately after the breach was repaired, where *K* = *D* without a valve effect and *K* = 2*D* − (*DP*/*Q*) with a valve effect (Figure 1b). In future models, the term *K* may be modified further to fit any baseline population post-breach.

In scenario 5, we used the following mathematical equation to determine *y*(*t*), the internal slug population at a steady state with a constant breach (e.g., an open tunnel or many small tunnels present in porous soil) throughout the experiment, and a valve effect in place (Figure 1c). This scenario was based on a question raised by farmers on the island of Hawaii, where the ground has porous gravel that creates pathways where slugs might tunnel under the barrier. The new term *T* represented the proportion of slugs crossing (entering and exiting) the tunnel (s) once they reached the perimeter (barrier circumference) of the tunnel; here, *T* is the same for entry and exit (no valve effect for the tunnel). The term *U* was the entrance and exit circumference of the tunnels (assumed to be the same). We defined *C* and *T* as the circumference of the barrier and barrier breach, respectively, *V* as the velocity of the slugs [18], *A* as the area of the protected plot, and *t* as time.

$$y\left(t\right)=D\frac{\left(CP+UT\right)}{\left(CQ+UT\right)}+Ke^{-\left(\frac{V}{A}\right)\left(CQ+UT\right)t}(\mathrm{Scenario}\;5)$$

In scenario 6, we modified *P* and *Q* to explore a repelling effect that changes the proportion of slugs that cross the barrier once encountered. To investigate this effect independent of a valve effect, we set *P/Q* = 1 and modified their values.

For all scenarios with valve effects, parameters *P* = 0.1 and *Q* = 0.9; these estimates were based on previous findings in which the ratio of slug densities in the experimental treatment to control plots at a steady state was 9:1, respectively [13]. The experimental data used to estimate the valve effect were taken from two field experiments conducted from May 2020 to February 2021 at a local organic farm in Kula, Hawaii [13]. Infested produce was reported by the farm owner, and surveys confirmed the presence of *D. reticulatum* at this site. Two field experiments were conducted over a period of 10 and 25 weeks, respectively. The first compared slug densities in a plot protected by a barrier with a valve mechanism (electricity) against two control plots protected by barriers without a valve mechanism (no electricity); the second control plot was included to confirm that there was no deterring effect of metal barrier materials on density. The second experiment compared slug densities in a protected and unprotected plot. Plots were approximately 6 m^2^ and treated with pellets at the start of each experiment. A barrier breach occurred in the first experiment that was repaired, which resulted in a temporary spike in the treatment plot density followed by a return to previous levels. In all models, slug velocity was set to *V =* 0.18 m/h; this estimate was generated by calculating the velocity (see Appendix A) using the median of an observed range of movement rates for *D. reticulatum* in a laboratory setting [12]. Placeholder values were used for all other parameter estimates at *t_0_*; the same values for these parameters were used in all models to test the relative effects of different scenarios on observed population densities.

## 3. Results

The lowest internal population density at steady state (as variable time *t* approached infinity) resulted from a valve effect (scenarios 2 and 4), with and without a single barrier breach, respectively (Figure 1a,b). In both scenarios, the internal population density approached a density of approximately 10% of the external population density. The second lowest internal population density resulted from a valve effect and constant breach (Figure 1c) of the barrier (scenario 5). In this scenario, the internal population density approached approximately 40% of the external population density at a steady state. The highest internal population density resulted from barriers without a valve effect (scenarios 1, 3, and 6), without and with a single barrier breach (Figure 1a,b), and with a repelling effect (Figure 1d), respectively. In these scenarios, the internal population density approached the same density of the external population at a steady state (i.e., the barrier had essentially no effect of reducing the internal population density of slugs); in other words, with no valve effect, it is only a matter of time before the internal and external population densities are the same at steady state.

In addition to predicting outcomes at a steady state, this model also elucidates key parameters that may determine the rate at which a steady state is reached. This model predicts that a steady state will be reached more rapidly at sites where slugs move at greater velocities, as barrier circumference increases, plot areas are made smaller, a greater proportion of slugs exit the protected plot (i.e., a strong valve effect), and travel via tunnels under the barrier increases (i.e., related to larger tunnel circumferences and proportions of slugs crossing).

## 4. Discussion

This study demonstrates how different barrier designs can lead to potentially very different outcomes for rat lungworm disease mitigation via control of slug carrier populations. We explore the underlying mechanisms affecting population densities of slug carrier species in different scenarios: in response to barriers with and without a valve effect, when barriers are breached and repair is both feasible and infeasible, and barriers with a repelling versus a valve effect.

A key finding of this study is that barriers with valve effects are essential to reduce and maintain lower densities of slugs at a steady state in protected areas. In the absence of a valve effect, the internal population density eventually approached the same value as the external population density; this outcome is predicted to occur regardless of whether the starting density of the internal population is lower (e.g., the internal plot is initially cleared of slugs) or higher (e.g., slugs invaded the internal plot) relative to the external population. With a valve effect, a repaired single breach was predicted to create an initial spike in the internal population density that eventually returned to the same internal density at a steady state as that in the absence of a breach. Left unrepaired, a constant breach reached a steady state density that was higher than when there was no breach (and a valve effect), but lower than when there was no valve effect (and a breach). Thus, an apparatus with a valve effect is predicted to sustainably reduce slug population densities, even when the efficiency of the barrier is reduced by breach events.

While repelling effects may delay the invasion of slugs into a protected area by reducing the rate of crossing a barrier, they do not produce the same outcomes as barriers with valve effects. Rather, barriers that employ only repelling effects do completely prevent the passage of slugs across that barrier [19] and are predicted to simply reduce the rate at which the densities of the protected plot and external populations approach the same value at a steady state (Figure 1d). In theory, barriers without valve effects that severely delay encroachment on a protected crop (e.g., on time scales comparable to a crop’s plant-to-harvest cycle) could have practical applications for maintaining lower slug densities prior to harvest. However, installing such barriers that employ only repelling effects would not maintain reduced slug densities at a steady state, resulting in higher densities within protected areas for additional crops planted later in the same growing season. Past barrier designs have commonly focused on the repelling effects of barrier materials, such as copper, to deter slugs [20]. While there has been some evidence that copper can exclude slugs if used in conjunction with a repellent [21,22], the anecdotal evidence for its usefulness for reducing slug densities is mixed [23]. The inconclusive findings of previous investigations of copper could be an artifact of sampling timing due to potentially high variation in density estimates obtained prior to reaching a steady state. However, despite limited evidence for deterrent materials, repelling and valve effects need not be mutually exclusive; plots protected by barriers containing a valve effect with a repelling effect may approach lower steady-state levels inversely proportional to the strength of the valve effect. However, without a valve effect, the internal and external densities are predicted to eventually approach the same value [23], providing support for the use of valve mechanisms for the long-term reduction of slug carriers in protected areas.

Parameters affecting the rate at which steady state is reached are of key interest to predict how rapid target outcomes (e.g., observed reductions in slug densities) may be achieved. We predicted a more rapid approach towards a steady state in systems with slug species that move at greater velocities, large ratios of barrier circumferences to plot areas, and a higher exit rate of slugs from the protected plot. In cases where there is a constant breach of the barrier, increased movements of slugs via tunnels would result in a more rapid approach to steady-state densities. These parameters highlight the importance of considering spatial, temporal, and species-specific factors when designing protective barriers. For example, in scenarios with porous soil and high opportunities for tunneling, slower-growing crops, and dominant slug species that move at faster rates (e.g., *P. martensi*), barriers relying on repelling effects only may be quickly overwhelmed, whereas barriers with valve mechanisms may offer a more effective solution for maintaining lower densities of slugs.

Additionally, factors challenging the model assumptions of homogenous spatial distributions and random movements of slugs within a site may affect the rate at which target outcomes at a steady state are achieved. Potentially critical factors contributing to site-specific, patchy spatial distributions of slugs [24] include density-dependent dispersion and nonlinear movement patterns [12], behavioral interactions [25], and seasonal variation in movement rates. Conditions that reduce encounter rates with the barrier (e.g., reduced slug velocities, repelling conspecific interactions near the barrier, and relatively lower external population densities due to patchiness) are predicted to increase the amount of time to reach a steady state. If a steady state, in which the population density of the protected plot is reduced by 90% relative to the external population, is a target outcome that is time-sensitive, these factors should be considered and tested prior to deploying this apparatus at scale in a given location. In future models, the velocity term may be refined to investigate the potential effects of the above factors on barrier interactions and the resulting rate at which target outcomes are reached. Future field experiments may confirm model predictions using larger plots to investigate the effects of this barrier apparatus at scale.

The simplified deterministic models used in this study did not include birth and death rates or general trends in slug movement direction (i.e., the models assume a random movement of slugs). The parameters for slug reproduction and death were excluded because of the relatively short time scale of slug dispersion compared to its life cycle; this process is supported for other systems in the spatial dynamic population literature, where species distribution occurs on shorter time scales relative to other population dynamics [26]. One generation of *P. martensi* is approximately five to six months [27], whereas, based on previously published field data, the time to reach a steady state is approximately 5 weeks [13]. The parameter estimate for the valve effect in this model was based on experimental field data [13], which accounted for potential competing effects beyond that of the valve effect (e.g., slug preference for higher quality habitat within protected plots). Such opposing effects would contribute to a more conservative estimate of the valve effect modeled in this study. In future models, densities may be modeled over multiple generations or include a modified velocity term (Appendix A) to account for slug habitat preference (e.g., whereby slugs are less likely to exit than enter the protected plot via the barrier).

In future experiments, the model predictions presented in this study could be tested using field experiments that measure changes in slug densities over time in response to breach events, repelling effects, and valve effects. Field and laboratory studies to test the effect of a valve mechanism against other slug species would also improve our ability to accurately predict species-specific outcomes. Additionally, alternative types of valves may improve upon the barrier design explored in this study [13] and could be tested using both field and laboratory experiments. The field experiment demonstrating a 90% reduction in slug densities used relatively small 6 m^2^ plots, which are much smaller than large-scale agricultural projects. While the densities of protected plots are predicted to approach a 90% reduction at steady state regardless of plot size, very large-scale plots may contain more nonuniform patches of slug densities and variable strengths of valve effects along the barrier. Additionally, site-specific factors may interact with plot size and barrier circumferences at these larger scales. Some key site-specific factors include weather conditions, slug nutritional state, attractants inside of the plot (e.g., crop type), conspecific interactions (e.g., slug–slug interactions and trails), and slug species. Stochastic models may be useful to account for greater variability in model predictions due to these factors. When the timing of a target outcome is important, in silico experiments would also be useful to determine optimal plot sizes for a given set of site-specific conditions. When possible, the predicted outcomes for different plot sizes should be confirmed prior to the deployment of this barrier apparatus in an agricultural setting.

Practical and effective measures of crop protection against rat lungworm disease gastropod carriers are an essential component to successfully manage the epidemic in Hawaii state, where *A. cantonensis* is broadly distributed and has the potential to expand its range to higher elevations due to warmer average temperatures caused by climate change [8], and annual case counts of this disease are rising [1]. More efficient barriers contributing to the long-term reduction of gastropod carrier densities may not only reduce rat lungworm disease risk, but also has potential applications for a wide array of other diseases transmitted by gastropods to humans (e.g., clonorchiasis, fascioliasis, fasciolopsiasis, opisthorchiasis, paragonimiasis, and schistosomiasis) [28]. Anecdotal observations in field and laboratory settings suggest that this apparatus is effective against snails as well, offering a potentially wide application of these barriers in reducing the densities of various terrestrial gastropods. The addition of valve mechanisms to slug barriers may also help to offset the existing multimillion-dollar costs of terrestrial gastropod-related crop damage in agricultural industries [29]. The apparatus explored in this study [13], which may primarily be employed by farmers, has been designed to be economically attainable at small scales. The cost of materials for the apparatus used in the field component of this project [13] is approximately USD 1944.00 to protect a one-square-hectare plot, including batteries and refugia to monitor changes in the internal slug density of the plot. The cost of materials would likely be reduced if purchased in larger quantities for larger farms, but it also offers a potentially feasible solution for smaller farms. These materials may be reused for future seasons, apart from the batteries. Such solutions not only provide farmers with a means to protect existing crops, but also to potentially grow more delicate crop species that are less resistant to slug herbivory (e.g., napa cabbage). Additionally, for organic farms, this apparatus provides a chemical-free solution to reduce pests’ damage to produce.

In conclusion, our findings support the use of valve mechanisms in barriers to rat lungworm disease slug carriers. This key design component is predicted to yield a long-term reduction in the population densities of slug carriers in protected areas. The use of effective protective barriers is essential, not only to mitigate disease risk, but to promote the economic and cultural welfare of farmers and local communities.

## Figures and Tables

**Table 1:** The effects of valves on the internal population density of organisms. Arrows represent the direction of movement of animals, red lines represent electric barriers, and shaded regions represent the protected plot inside of the slug barrier.

Type	Valve	No Valve
	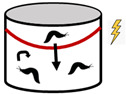	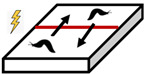
Direction	Entry < Exit	Entry = Exit
Effect on Internal Density	Decrease	None
Distinguishing Factors	Gravity, Electricity	Electricity

**Figure A1:**
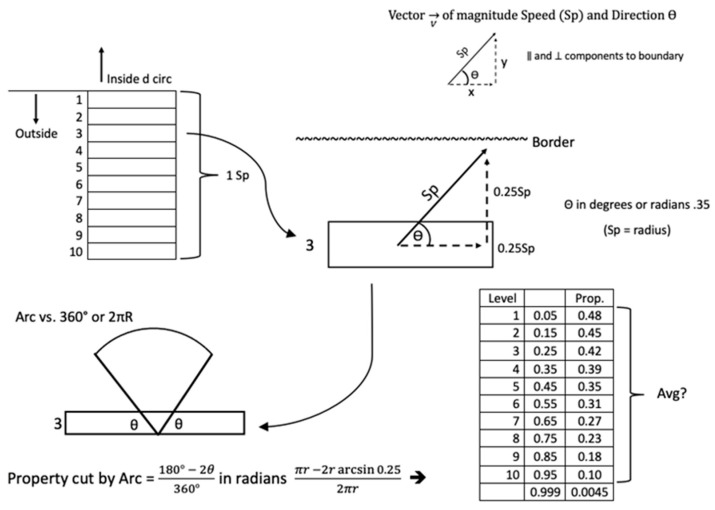
Calculation of the proportion of slugs approaching a barrier based on their initial distance from the barrier and velocity (i.e., speed and direction).

**Figure A2:**
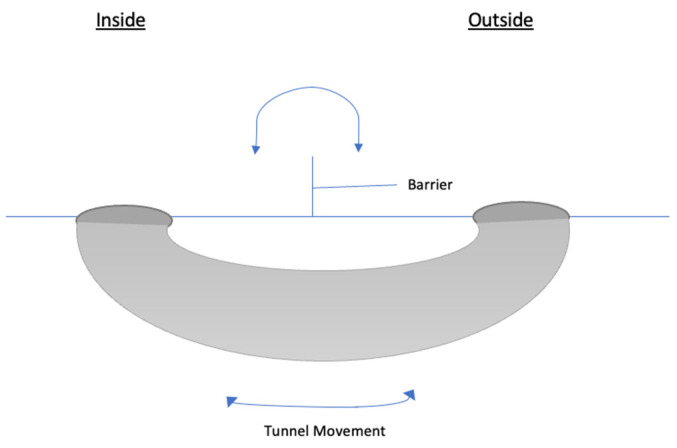
Diagram of the tunnel (constant breach) in which slugs can move between the inside and outside of a plot protected by a barrier.

## Appendix A

A more detailed explanation of the mathematical expression will be given here. We will not cover how the solution (integration) for variable *Y* is derived from the differential equation, which can be found in standard references for ordinary differential equations (ODE). Furthermore, we will not “double-check” to show that this solution for *Y* does, in fact, fit the ODE. Instead, this appendix will describe in more detail the parameters of the math expression in terms of the physical slug and garden setting. Notably, while the basic equation tracks the number of slugs (abundance), we would like an expression for slug densities (number per unit area). As we describe parameters and how they fit into the equations, it is very useful to keep track and be cognizant of the units to provide an intuitive sense. For example, exponential powers should be in terms of pure numbers (except for the scalar of time) if the independent variable *x* were to represent time.

The approach to examine the mathematics of the model is to first set up an intuitive tally balancing the change (difference over time, differential) in the number of slugs in the garden. Over an interval of time, this change will be the number entering minus the number leaving. Assume that movement dominates the changing numbers (such as birth, deaths, and predation). Then, we examine the parameters of the equation (the physical aspects of slug movement in our setting) to express the change in slug density rather than numbers. We isolate terms that are time-dependent and those that are not. Based on the type of differential equation, we can solve for the value of density itself. This solution will incorporate the initial parameters and introduce one new parameter, the initial baseline density.

Based on our previously published laboratory and field experiments (13) with this slug/snail barrier, we start with a general equation #1, which balances the inward and outward movements of the number of slugs (abundance). From this first expression, we will determine a solution as shown in equation #2, as well as look at two separate circumstances added onto equation #1: a breach that is fixed after a fixed number of slugs enter and exit through, as shown in equation #3, and a breach which cannot be fixed but allows slugs to enter and exit at a constant rate as shown in equation #4.

Equation #1 (Differential Equation for Slug Abundance)

The change in the number of slugs per unit of time is equal to the number entering (across the barrier) minus the number exiting. There are no alternate pathways (breaches).

$$\frac{dN}{dt}=K_{1}-K_{2}$$

Where: *N* is the total number (or abundance) of slugs in the garden (number).

Where:

$$K_{1}=\left(C\right)\left(V\right)\left(pOut\right)\left(ProEnt\right)$$

Where:

*C* is the circumference of the garden (cm).

*V* is the slug velocity vector of both speed and direction to reach the barrier (cm per time).

*pOut* is outside slug density (number per cm^2^).

*ProEnt* is the proportion of slugs that enter across the barrier once they have reached it from the outside.

Where:

$$K_{2}=\left(C\right)\left(V\right)\left(pIn\right)\left(ProEx\right)$$

Where:

*C* and *V* are defined as above.

*pIn* is the inside slug density, dependent on time (number per cm^2^).

*ProEx* is the proportion of slugs that exit across the barrier once they have reached it from the inside.

Notes:

1. The outside slug density is assumed to be constant, whereas the inside density depends on time (the time from when the barrier is activated with some initial baseline population). This initial baseline population will not appear in the differential equation and will only appear later (after integration) when one solves *N* itself. Mathematically, it will be a constant of integration that can be set to match the starting baseline internal slug population;

2. It was reported in the laboratory experiments of the original publication (13) that for a vertical barrier, as slugs approached from the top (exiting the garden barrier), they could retreat (crawl back up) while others “crossed” the barrier, either falling over it or crawled across it (very rare). When slugs approached from the bottom (entering), none climbed across the barrier; they either crawled back down or fell back due to the electric shock. Equation #1 allows for two different proportions for entry and exit, and “crossing” the barrier can occur by falling or crawling over it. With the barrier set up outside the garden, the falling movements favor exiting over the entry;

3. Here is a detailed explanation for the slug velocity *V*; see Figure A1 The velocity vector has two components: speed (*Sp,* which is the magnitude) and direction (*θ*). The total circumference can be divided into smaller lengths (approaching infinitely small segments as we apply calculus to tally the total number of slugs reaching the entire circumference). Corresponding to each segment, there is an area of flux, defined as the area in which all slugs have the potential to reach the barrier in time *t*. The distance of the upper border of the flux area is set to the distance calculated by a slug moving at speed *Sp* for a time *t*. This flux area excludes slugs that are too far away. In this flux area, let all the slugs have an average speed, *Sp*.

To get a notion of the importance of direction (*θ*) to reach the barrier, at this distance, only slugs traveling in the direction perpendicular to the circumference segment will reach the barrier. At the extreme limit of the distance, only 1/360 will be going in the right direction to reach the barrier. For the slugs at the barrier, half of them will have the correct direction (180/360) to reach it, while the other half will move away.

Figure A1 shows a typical flux area along the circumference where slugs enter the garden approaching it from the outside. A similar diagram can be drawn in reverse, where slugs exit the garden by approaching it from the inside. The flux area is broken up into 10 levels, each with midpoint perpendicular distances corresponding to *Sp* from closest to farthest: 0.05 *Sp*, 0.15 *Sp*, 0.25 *Sp*, …, 0.95 *Sp*. For example, looking at level 3, only slugs with enough perpendicular direction will reach the border at 0.25 *Sp*; based on trigonometry, the limit will be set by the angle *θ*, whose sine is 0.25. All slugs traveling more “vertically” than *θ* off the horizontal axis will reach the border; therefore, the proportion of slugs moving in the right direction will equal the following: (180 − 2*θ*)/360 = 42%. The figure shows another column with the proportion of slugs moving in the right direction for each stratum. Across all strata of the flux area, the average of the slugs moving in the right direction is 32%. Alternatively, mathematically one could get an exact proportion by integrating across infinitely small layers with their corresponding proportions bounded by these angles (it is good to convert the angles in radians to avoid switching positive and negative values of sine as one crosses quadrants).

If slug motion is random in all directions, for any given average speed of slugs, only about 32% of the slugs are moving in the right direction to reach the barrier. Where *Sp* is the average speed of the slug, and its unit is cm/time unit. The unit of time is a scalar to be chosen for the entire equation (i.e., seconds, minutes, hours, days, weeks, etc.). We assume the random direction (*θ*) of the slug’s movement for entering and exiting the garden. For the same value *Sp*, refinement to the model can be made so that if there is an attractant in the garden, there will be a non-random, higher percentage of slugs moving in the direction to reach the barrier from the outside and the opposite effect for the slugs already inside the garden;

4. The number of slugs in the garden *N* is the fixed area of the garden (*A*) multiplied by the inside slug density (*pIn*). The differential equation #1 can be rewritten to convert *N* into density and to isolate the time-dependent variable, which is the inside slug density *pIn*.

Where:

$$\frac{dpIn}{dt}=\frac{K_{1}}{A}-\frac{K_{2^{\prime }}}{A}\;\left(pIn\right)\;$$

Where: *A* is the area of the garden (cm^2^).

From the form of the above differential equation of the time-dependent density, the solution for this differential would be:

*K*_2_′ = *K*_2_/*pIn*

*pIn* = (*K*_1_/*A*)/(*K*_2_′/*A*) + *C*3 × e^−(*K*^_2_^′/*A*)*t*^

The new constant of integration *C*3 is introduced and can be used to “set” the initial baseline *pIn* levels (when *t* = 0). Its units are the same as the density and number per cm^2^.

Equation #2

To better appreciate the parameters of the mathematical model and to track the unit, let us substitute the parameters for the dummy variables *K*_1_, *K*_2_, and *K*_2_′, as demonstrated here:

$$\begin{array}{ll} pIn & =\frac{\left(\frac{\left(C\right)\left(Sp\right)\left(0.32\right)\left(ProEnt\right)\left(pOut\right)}{A}\right)}{\left(\frac{\left(C\right)\left(Sp\right)\left(0.32\right)\left(ProEx\right)}{A}\right)}+C3e^{\frac{-\left(C\right)\left(Sp\right)\left(0.32\right)\left(ProEx\right)t}{A}}\\ & =\frac{\left(ProEnt\right)\left(pOut\right)}{ProEx}+C3e^{\frac{-\left(C\right)\left(Sp\right)\left(0.32\right)\left(ProEx\right)t}{A}} \end{array}$$

At the steady state when *t* gets very large, the second term approaches zero, and *pIn*/*pOut* = *ProEnt*/*ProEx*. For example, when it is 10 times easier to exit than to enter, the internal slug density will be one-tenth of the outside density. This difference between the proportion that enters versus exits is what we term the “valve” effect. It is similar to the valve effect of funnel fish traps, except that the funnel is set to concentrate fish inside the trap rather than outside. Alternatively, at a steady state, the internal and external densities will be equal if the chances of entering and exiting are the same. This is true even for very effective barriers without a valve effect, for example, the same electric-barrier system (blocking 90% of slugs) but laid horizontally without the valve effect of shock + gravity, or even barriers with repelling effects, such as copper.

Next, the equation shows the factors which affect how quickly the steady state will be reached. Looking at the components of the factors of the negative exponent, we see that an increased garden circumference, greater speed of slug movement, a high proportion of slugs exiting, and a decreased garden area will approach a steady state faster. Furthermore, the exponent is a pure number with the time scale matching the time units of the slug speed.

Finally, the value of *C*3 is determined algebraically based on whatever we choose as the baseline population density in the garden (when *t* = 0).

As an example of the above principles, suppose we do not attempt any baseline “one-time” clean out of the slugs in the garden. This population will then approach a steady-state level depending on the valve effect (regardless of the intrinsic repelling effect of the material). How quickly this occurs depends on the speed of the slugs, the circumference, and the area of the garden.

Equation #3: Effects of a One-Time Breach

Building upon equations #1 and# 2 (using baseline *pIn* = 0, and *ProEx*/*ProEnt* = 9:1 valve effect, same slug average speed and garden *C* and *A),* the differential equation of slug abundance during a one-time breach will be the following which addresses the entry and exit via the one-time breach:

*dN*/*dt* = (*K*_1_ + *B_ent_*) − (*K*_2_ + *B_ex_*)

Where:

*B_ent_* is the number of slugs entering the garden via the breach over time (number per time unit)
*B_ex_* is the number of slugs exiting the garden via the breach over time (number per time unit)

The conversion of the equation to track slug densities is similar to what was done for equation #1 but introduces the effects of the breach. Immediately after the breach is fixed, we determine the final endpoint for the internal density, *pIn*. Moving forward after the breach is fixed, this endpoint becomes the new starting point that follows the rules of equation #2, but now with a new non-zero starting point. This principle of using equation #2 but with a new starting point is very useful since it does not really depend on what the breach is. For example, it might not be a pass-through through which slugs enter and exit, but simply a one-time breach of slugs, say slug-infested compost dumped into the garden. Thus, the method of handling slug densities after the breach has ended is nothing more than redefining a baseline slug density and proceeding with equation #2. Additionally, as we showed previously, this initial starting need not be zero and will approach the steady state through the dynamics of the model depending on various parameters. This starting point moving forward after the breach will determine the value *C*3.

For readers who laboriously tallied parameters during the breach to reach the endpoint density, your work will not go unrewarded. The next situation is a permanent, unrepaired breach, which is nothing more than a description of the model during the breach before it was repaired.

Equation #4: Effects of a Constant Breach

Finally, we address the concern of an “irreparable” tunneling network of rocky, porous ground through which slugs move. Mathematical models should be specific to the nature of the breach. However, if breaches can be quickly repaired (i.e., in our field experiment, the breaches were detected and repaired within 3 days), we have the luxury of moving forward with a no-breach model, with the residual breach effect incorporated by a new baseline starting point.

To highlight the principles of a continuous breach without oversimplification, we assume a similar setting with similar parameters as that described in equations #1 and #2: garden circumference, garden area, slug speed, the random direction of movement, valve effect (9:1), baseline *pIn* (zero). We add in the tunneling through the ground as our constant breach. To keep things simple, passing through the tunnel will follow the rules of passing through the barrier. The tunnel entrance(s) must be approached according to the rules of slug density, flux, and velocity. Of the slugs that reach the tunnel circumferences, there is a proportion that will pass through in either direction (entering or exiting). Assuming there is no valve effect on the tunnel, of those slugs that approach the tunnel entrance(s), the proportion of those entering is the same as those exiting. Furthermore, the tunnel option is treated as an alternative to the barrier: slugs will either choose to pass through the barrier or tunnel, not both.

The assumptions for the barrier are that the internal population density is 0 at baseline, the valve effect is 9:1, and there are no repaired breaches. The assumptions for the tunnel are that internal population density is 0 at baseline, there is no valve effect, and the tunnel effect is proportional to the circumference of the tunnel opening as opposed to its volume. The cumulative effect of multiple tunnels can be represented in a single term where circumferences of multiple tunnels are added together.

The differential equation is the net change in the slug abundance: the number entering minus the number exiting. Slugs can pass (enter and exit) through the tunnel or through the barrier. It is similar to equation #1: *dN*/*dt* = *K*_1_ − *K*_2_, but now the entry rate *K*_1_ and exit rate *K*_2_ are through the barrier or the tunnel.

*dN*/*dt* = (*K*_1*B*_ + *K*_1*T*_) − (*K*_2*B*_ + *K*_2*T*_)

Where:

*K_1B_* is the rate of slugs entering via barrier (number per unit of time).

*K_1T_* is the rate of slugs entering via tunnel (number per unit of time).

*K_2B_* is the rate of slugs exiting via barrier (number per unit of time).

*K_2T_* is the rate of slugs exiting via tunnel (number per unit of time).

The following equation is similar to equation #2, where we converted it to internal density and isolated the parameter of *pIn* because it is time-dependent:

$$\frac{dpIn}{dt}=\frac{\left(K_{1B}+K_{1T}\right)}{A}-\frac{\left(K_{2B^{’}}+K_{2T^{’}}\right)\left(pIn\right)}{A}$$

The solution for density at a steady state will be:

$$pIn=\frac{\left(\frac{\left(K_{1B}+K_{1T}\right)}{A}\right)}{\left(\frac{\left(K_{2B^{\prime }}+K_{2T^{\prime }}\right)}{A}\right)}$$

$$K_{2B^{’}}+K_{2T^{\prime }}=\frac{\left(K_{2B}+K_{2T}\right)}{pIn}$$

For variables *K*_2*b*′_ and *K*_2*T*_, please refer to the previous definition of *K*_2′_.

Filling out the parameters for the values of *K*, we have:

$$\begin{array}{ll} K_{1T} & =\left(CT\right)\left(Flux\right)\left(pOut\right)\left(ProT\right)\\ & =\left(CT\right)\left(V\right)\left(pOut\right)\left(ProT\right)\\ & =\left(CT\right)\left(Sp\right)\left(0.32\right)\left(pOut\right)\left(ProT\right) \end{array}$$

Where:

*CT* is the tunnel circumference (equal on both tunnel ends).

*ProT* is the proportion of slugs crossing via the tunnel.

Similarly,

$$K_{2T}=\left(CT\right)\left(Sp\right)\left(0.32\right)\left(pIn\right)\left(ProT\right)$$

Converting to the internal slug density,

$$\frac{dpIn}{dt}=\frac{1}{A}\left(Total\;rate\;enter-Total\;rate\;exit\right)$$

With the isolation of variable *pIn*,

$$\frac{dpIn}{dt}=C_{1}-C_{2}\left(pIn\right)$$

Where: *C*_1_ and *C*_2_ are placeholder variables:

$$\;\;\;\;C_{1}=\frac{1}{A}\left(pOut\right)\left(Sp\right)\left(0.32\right)\left(\left(C\right)\left(ProEnt\right)+\left(CT\right)\left(ProT\right)\right)$$

$$C_{2}=\frac{1}{A}\left(Sp\right)\left(0.32\right)\left(\left(C\right)\left(ProEx\right)+\left(CT\right)\left(ProT\right)\right)$$

Notice that the units of *C*_1_ and *C*_2_ differ by density unit. The unit of *C*_1_ is number/(cm^2^ × unit time). The unit of *C*_2_ is 1/unit time.

Solving for *pIn*,

$$pIn=\frac{C_{1}}{C_{2}}+C3e^{-C_{2}t}$$

At a steady state, the second term of the right side of the above equation approaches 0, leading to the following equation:

$$\frac{pIn}{pOut}=\frac{\left(\frac{1}{A}\right)\left(Sp\right)\left(0.32\right)\left(Z_{1}\right)}{\left(\frac{1}{A}\right)\left(Sp\right)\left(0.32\right)\left(Z_{2}\right)}$$

Where: *Z*_1_ and *Z*_2_ are placeholder variables such that:

$$Z_{1}=\left(C\right)\left(ProEnt\right)+\left(CT\right)\left(ProT\right)\;Z_{2}=\left(C\right)\left(ProEx\right)+\left(CT\right)\left(ProT\right)$$

*Z*_1_/*Z*_2_ is the ratio of the inside density to the outside density at a steady state. This ratio has the form:

$$\frac{a+c}{b+c}$$

When *c* is large (i.e., a very large tunnel effect) with respect to *a* and *b*, the internal population density approaches the external population density at a steady state (in other words, as if there is no barrier).

When *c* is small (i.e., a very small tunnel effect) with respect to *a* and *b*, the internal population density approaches the population density with a valve effect at a steady state (in other words, as if there is no tunnel).

To explore how quickly a steady state is reached with a tunnel effect, investigate the exponent term above. Equilibrium is predicted to be approached faster with increasing speed, barrier circumference, tunnel circumference, the proportion of slugs exiting via the barrier, the proportion of slugs exiting via the tunnel, and decreasing garden area.

## References

[1] Cowie R.H., Ansdell V., Dunavan C.P., Rollins R.L. (2022). Neuroangiostrongyliasis: Global spread of an emerging tropical disease. Am. J. Trop. Med. Hyg..

[2] Hwang K.P., Chen E.R. (1991). Clinical studies on *Angiostrongyliasis cantonensis* among children in Taiwan. Southeast Asian J. Trop. Med. Public Health.

[3] Jitpimolmard S., Sawanyawisuth K., Morakote N., Vejjajiva A., Puntumetakul M., Sanchaisuriya K., Tassaneeyakul W., Tassaneeyakul W., Korwanich N. (2007). Albendazole therapy for eosinophilic meningitis caused by *Angiostrongylus cantonensi*s. Parasitol. Res..

[4] Ansdell V., Kramer K.J., McMillan J.K., Gosnell W.L., Murphy G.S., Meyer B.C., Blalock E.U., Yates J., Lteif L., Smith O.A. (2021). Guidelines for the diagnosis and treatment of neuroangiostrongyliasis: Updated recommendations. Parasitology.

[5] Kim J.R., Wong T.W., Curry P.A., Hayes K.A., Cowie R.H. (2019). Modelling the distribution in Hawaii of *Angiostrongylus cantonensis* (rat lungworm) in its gastropod hosts. Parasitology.

[6] Hollingsworth R.G., Howe K., Jarvi S.I. (2013). Control measures for slug and snail hosts of *Angiostrongylus cantonensis*, with special reference to the semi-slug *Parmarion martensi*. Hawai’i J. Med. Public Health.

[7] Qvarnstrom Y., Bishop H.S., da Silva A.J. (2013). Detection of rat lungworm in intermediate, definitive, and paratenic hosts obtained from environmental sources. Hawai’i J. Med. Public Health.

[8] Kim J.R., Hayes K.A., Yeung N.W., Cowie R.H. (2014). Diverse gastropod hosts of Angiostrongylus cantonensis, the rat lungworm, globally and with a focus on the Hawaiian Islands. PLoS ONE.

[9] Yeung N.W., Kim J.R., Hayes K.A. (2018). Rat lungworm (*Angiostrongylus cantonensis*) in Hawai’i: Updated host gastropod records and distributions on Maui. Bish. Mus. Occas. Pap..

[10] Cowie R.H. (2013). Pathways for transmission of angiostrongyliasis and the risk of disease associated with them. Hawai’i J. Med. Public Health.

[11] Fogarty M.J., Addison J.T. (1997). Modelling capture processes in individual traps: Entry, escapement, and soak time. ICES J. Mar. Sci..

[12] Ellis J., Petrovskaya N., Forbes E., Walters K.F.A., Petrovski S. (2020). Movement patterns of the grey field slug (*Deroceras reticulatum*) in an arable field. Sci. Rep..

[13] Pang L., Coppolo C., Hauptman S. (2022). An effective barrier to prevent crop contamination by slug vectors of *Angiostrongylus cantonensis*. Am. J. Trop. Med. Hyg..

[14] Arditi R., Tyutyunov Y., Morgulis A., Govorukhin V., Senina I. (2001). Directed movement of predators and the emergence of density-dependence in predator-prey models. Theor. Popul. Biol..

[15] Shaw A.K., White L.A., Michalska-Smith M., Borer E.T., Craft M.E., Seabloom E.W., Snell-Rood E.C., Travisano M. (2021). Lessons from movement ecology for the return to work: Modeling contacts and the spread of COVID-19. PLoS ONE.

[16] Azimzade Y. (2022). Invasion front dynamics of interactive populations in environments with barriers. Sci. Rep..

[17] R Core Team (2022). R: A language and environment for statistical computing. R Foundation for Statistical Computing.

[18] Lai J.H., del Alamo J.C., Rodriguez-Rodriguez J., Lasheras J.C. (2010). The mechanics of the adhesive locomotion of terrestrial gastropods. J. Exp. Biol..

[19] Symondson W.O. (1993). Chemical confinement of slugs: An alternative to electric fences. J. Molluscan Stud..

[20] Schuder I., Port G., Bennison J. (2003). Barriers, repellents and antifeedants for slug and snail control. Crop Prot..

[21] Turke M., Heinze E., Andreas K. (2010). Seed consumption and dispersal of ant-dispersed plants by slugs. Oecologia.

[22] Kheirodin A., Damavandian M.R., Sarailoo M.H. (2012). Mineral oil as a repellent in comparison with other control methods for citrus brown snail, *Caucasotachea lencoranea*. Afr. J. Agric. Res..

[23] Watz J., Nyqvist D. (2021). Artificial barriers against arionid slug movement. Crop Prot..

[24] Forbes E., Back M.A., Brooks A., Petrovskaya N.B., Petrovskii S.V., Pope T.W., Walters K.F.A. (2020). Locomotor behavrio promotes stability of the patchy distribution of slugs in arable fields: Tracking the movement of individual *Deroceras reticulatum*. Pest Manag. Sci..

[25] Petrovskii S., Ellis J., Forbes E., Petrovskaya N., Walters K.F.A. (2022). A predictive model and a field study on heterogenous slug distribution in arable fields arising from density dependent movement. Sci. Rep..

[26] Potts J.R., Lewis M.A. (2019). Spatial memory and taxis-driven pattern formation in model ecosystems. Bull. Math. Biol..

[27] Hamilton L.J., Tagami Y., Kaluna L., Jacob J., Jarvi S.I., Follett P. (2021). Demographics of the semi-slug *Parmarion martensi*, an intermediate host for *Angiostrongylus cantonensis* in Hawai’i, during laboratory rearing. Parasitology.

[28] Lu X., Gu Q., Limpanont Y., Song L., Wu Z., Okanurak K., Lv Z. (2018). Snail-borne parasitic diseases: An update on global epidemiological distribution, transmission interruption and control methods. Infect. Dis. Poverty.

[29] Jiang X., Zheng P., Soto I., Haubrock P.J., Chen J., Ji L. (2022). Global economic costs and knowledge gaps of invasive gastropods. Ecol. Indic..

